# Understanding how Lewis acids dope organic semiconductors: a “complex” story†‡

**DOI:** 10.1039/d1sc01268a

**Published:** 2021-04-19

**Authors:** Pablo Simón Marqués, Giacomo Londi, Brett Yurash, Thuc-Quyen Nguyen, Stephen Barlow, Seth R. Marder, David Beljonne

**Affiliations:** Laboratoire MOLTECH-Anjou, UMR CNRS 6200, UNIV Angers, SFR MATRIX 2 Bd Lavoisier 49045 Angers Cedex France; Laboratory for Chemistry of Novel Materials, University of Mons Place du Parc, 20 7000 Mons Belgium david.beljonne@umons.ac.be; Center for Polymers and Organic Solids, Department of Chemistry & Biochemistry, University of California Santa Barbara California 93106 USA; Center for Organic Photonics and Electronics, School of Chemistry and Biochemistry, Georgia Institute of Technology Atlanta Georgia 30332-0400 USA

## Abstract

We report on computational studies of the potential of three borane Lewis acids (LAs) (B(C_6_F_5_)_3_ (BCF), BF_3_, and BBr_3_) to form stable adducts and/or to generate positive polarons with three different semiconducting π-conjugated polymers (PFPT, PCPDTPT and PCPDTBT). Density functional theory (DFT) and time-dependent DFT (TD-DFT) calculations based on range-separated hybrid (RSH) functionals provide insight into changes in the electronic structure and optical properties upon adduct formation between LAs and the two polymers containing pyridine moieties, PFPT and PCPDTPT, unravelling the complex interplay between partial hybridization, charge transfer and changes in the polymer backbone conformation. We then assess the potential of BCF to induce p-doping in PCPDTBT, which does not contain pyridine groups, by computing the energetics of various reaction mechanisms proposed in the literature. We find that reaction of BCF(OH_2_) to form protonated PCPDTBT and [BCF(OH)]^−^, followed by electron transfer from a pristine to a protonated PCPDTBT chain is highly endergonic, and thus unlikely at low doping concentration. The theoretical and experimental data can, however, be reconciled if one considers the formation of [BCF(OH)BCF]^−^ or [BCF(OH)(OH_2_)BCF]^−^ counterions rather than [BCF(OH)]^−^ and invokes subsequent reactions resulting in the elimination of H_2_.

## Abstract 2

Here we report on DFT calculations investigating the mechanistic aspects in doping organic semiconductors by the use of Lewis acids. Our results highlight the role played by the formation of diboron-containing bridged anions in the doping mechanism.

## Graphical abstract

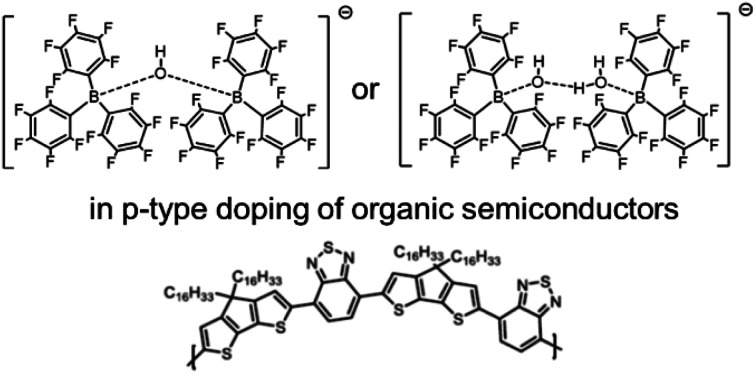

## Introduction

Molecular doping^1–6^ is a paramount topic in the organic semiconductor community, where it can enhance charge-carrier density and therefore electrical conductivity, improve charge injection and lower contact resistance, or increase charge mobility thanks by filling traps. The most straightforward approach to p- or n-doping is to use simple one-electron oxidants or reductants that react with the semiconductor to generate radical cations or anions (positive or negative polarons). A less intuitive approach to doping involves Lewis acids (LAs), notably tris(pentafluorophenyl)borane (BCF). Depending on the nature of the semiconducting polymers, LAs either effectively act as p-dopants or form Lewis Acid–Base (LAB) adducts.^7^ The aim of this computational study is to give insight into these two types of reactivity.

A decade ago, it was demonstrated that LAs can form physical complexes with semiconducting π-conjugated polymers,^8^ a process driven by the interaction between the empty p-orbitals of the centrally electrophilic boron atom in the LA and the electron lone pair of a Lewis base (LB) site on the polymer, such as a pyridyl nitrogen. The formation of a new stable covalent bond yields a LAB adduct with a specific fingerprint in optical absorption^9^ and increased charge carrier density with respect to the unbound polymer,^10–12^ representing a means of post-synthetic engineering.^13^ More specifically, alternating donor–acceptor conjugated copolymers, where the acceptor moiety is pyridylthiadiazole (PT), are able to strongly coordinate LAs, such as BCF, likely resulting in partial ground-state charge transfer (CT). The interaction with BCF has been shown to translate into a red-shifted onset in optical absorption of the organic semiconductor by ∼0.3 eV, a shift primarily due to the effect of the electron-withdrawing LA moiety on the electron affinity in presence of the LA itself.^13^

Rather unexpectedly, BCF can also act as an apparent oxidant. Indeed, in the late 1990s Doerrer and Green^14^ demonstrated that BCF – either when used intentionally as its 1 : 1 water complex BCF(OH_2_), which is a strong Brønsted acid, or in the presence of adventitious water – can behave as a strong oxidant, converting metallocenes (MCp_2_, M = Fe, Cr, Co) to the corresponding MCp_2_^+^. They considered that oxidation likely proceeded by protonation of MCp_2_ by BCF(OH_2_), followed by elimination of H_2_ from two MCp_2_H^+^ ions. Interestingly, the products they obtained did not contain the simple [BCF(OH)]^−^ anion (which is known and crystallographically characterized in other contexts^15^), but rather either [BCF(OH)BCF]^−^ or [BCF(OH)(OH_2_)BCF]^−^ anions. More recently, the oxidizing characteristics of BCF have been rediscovered in the context of the p-doping of organic semiconductors.^16^ BCF behaves as a strong oxidant, consistent with the findings of Doerrer and Green, but inconsistent with a simple one-electron transfer from polymer to BCF. It has been observed that BCF is reduced to the unstable radical anion at *ca.* −1.7 to −1.8 V *versus* ferrocene,^17^ whereas polymers that have been doped by BCF are oxidized at potentials comparable to, or more positive than, ferrocene, indicating that such an electron transfer would be highly endergonic. Thus, BCF(OH_2_), or other BCF(OH_2_)_*n*_ adducts, which are strong Brønsted acids and are formed by the hygroscopic BCF (unless water is scrupulously excluded), are thought to be the likely oxidant, if not by a direct one-electron transfer manner. In some cases, the use of BCF may be desirable relative to the very widely used 2,3,5,6-tetrafluoro-7,7,8,8-tetracyanoquinodimethane (F_4_TCNQ), due to its solubility in organic solvents, its lower volatility, and its ability to dope molecular materials with a relative high ionization potential (∼5.8 eV).^11,12,18^ On the other hand, other p-dopants that act as clean one-electron-oxidants may be more predictable in their behaviour as a consequence of their more straightforward chemistry.^19,20^ In any case, Yan *et al.* have successfully used BCF as molecular dopant in a donor:acceptor planar heterojunction device structure and found that LA doping plays a synergistic role in changing the opto-electronic properties and nano-morphology of the blends leading to improved device performances, even at low doping concentration.^21–23^

Consistent with the work of Doerrer and Green,^14^ it has been suggested that some particular polymers like poly-cyclopentadithiophene-benzothiadiazole (PCPDTBT) can be also oxidized by BCF(OH_2_) *via* an initial protonation step of the cyclopentadithiophene (CPDT) unit in the polymer backbone. In ref. 16 it was proposed that the resulting protonated, positively charged, polymer chain would undergo an increase in electron affinity (compared to the pristine polymer) large enough to prompt an electron transfer from another, pristine, polymer chain (or chain section), resulting in the presence of *two* radical species, *i.e.*, a neutral “protonated radical” and a radical cation (positive polaron). Continuous-wave electron-nuclear double resonance (ENDOR) spectroscopy affords a spectrum that is consistent with the presence of both radicals; specifically, a structureless spectrum is observed similar to what is expected for the “protonated radical”, while the polaron is expected to contribute a much less intense structured pattern. However, in a later work on p-doping of poly(3-hexylthiophene) (P3HT), Arvind *et al.* could observe only the radical cation using high-resolution electron paramagnetic resonance (EPR) spectroscopy, suggesting either the “protonated radical” does not form or that it is unstable against further chemical reactions.^24^ In particular, H_2_ elimination, as previously invoked in the contexts of both metallocene oxidation by BCF(OH_2_) and spiro-OMeTAD p-doping by HN(SO_2_CF_3_)_2_ (another strong Brønsted acid),^25^ has been suggested to play a paramount role, but to our knowledge formation of H2 has yet to be observed directly.

**Fig. 1:**
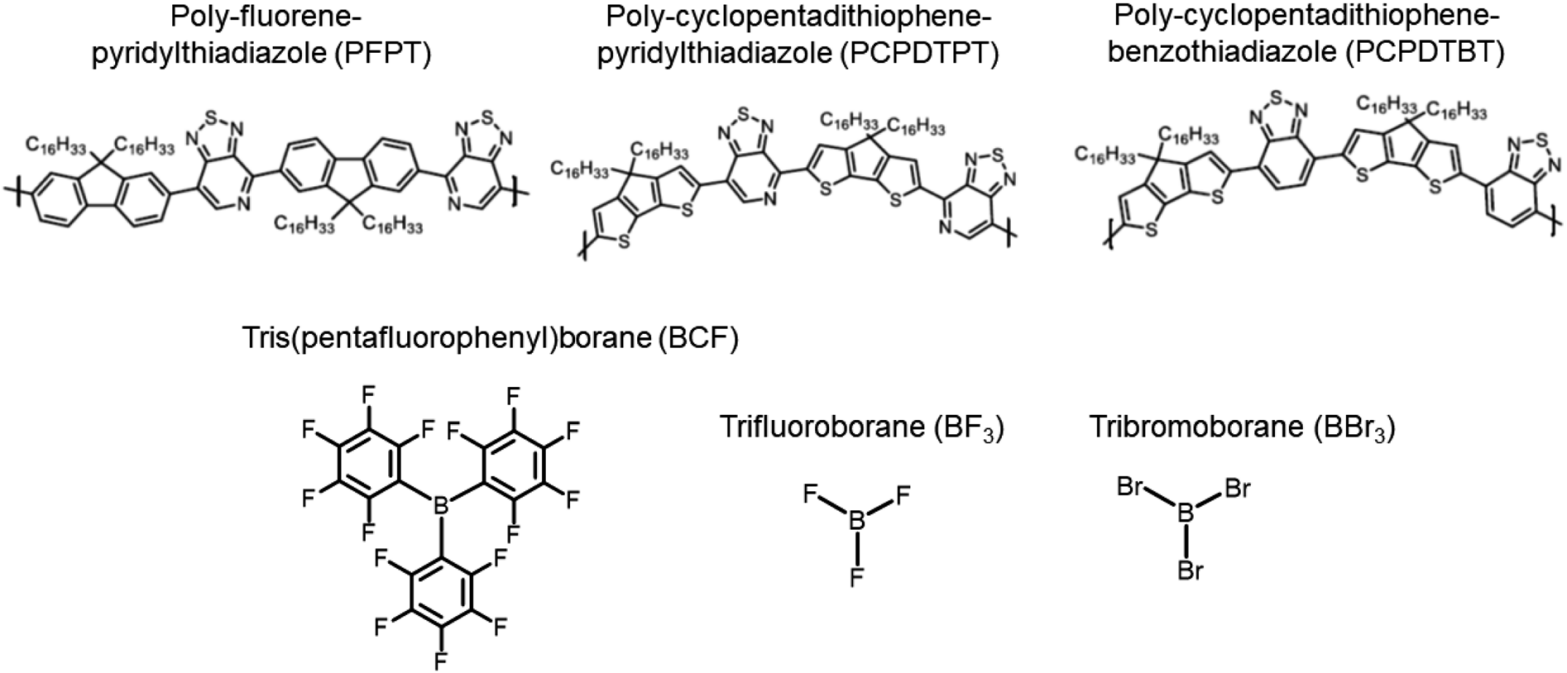
Chemical structures of the investigated polymers (top) and Lewis acids (LAs) (bottom).

A comprehensive description of how LAs interact with semiconducting π-conjugated polymers is currently lacking. Here, we report on state-of-the-art calculations investigating the potential of three boron-based LAs to either form physical complexes or undergo chemical reactions involving one-electron oxidation of the semiconductor with three different π-conjugated polymers (Fig. 1). Using density functional theory (DFT) and time-dependent DFT (TD-DFT) calculations based on optimally tuned (OT) range-separated hybrid (RSH) functionals,^26,27^ we first analyse the structural, energetics, and optical signature of ground-state complexes formed between three LAs and poly-fluorene-pyridylthiadiazole (PFPT) and poly-cyclopentadithiophene-pyridylthiadiazole (PCPDTPT) tetramers, finding good agreement with experiment and highlighting the factors affecting the changes in optical absorption. Though there is clear experimental evidence that LAs are able to dope some polymer semiconductors, the mechanistic aspects of the doping have not been elaborated yet. We thus move on in investigating the doping mechanisms of a PCPDTBT tetramer by BCF(OH_2_) from first-principles. This involves identifying the most likely protonation sites and assessing the energetics of previously proposed reactions. Our results show that those are highly endergonic, mostly due to the thermodynamically unfavourable protonation to form [BCF(OH)]^−^, thus ruling out all proposed mechanistic scenarios proposed in the literature. Capitalizing on the seminal work by Doerrer and Green, we instead consider reactions leading to the formation of larger complex anions, as observed in the context of metallocene oxidation.^14^ Remarkably, we then find that the resulting protonated PCPDTBT chains can undergo moderately endergonic reactions when eliminating H_2_ to produce a single spin-carrying charged species.

## Methods

Gas-phase ground-state equilibrium geometries of two representative tetramers, PFPT and PCPDTPT, were obtained by performing DFT optimization at the RSH functional level of theory, using the exchange-correlation *ω*B97X-D functional^28^ and the 6-31G(d,p) split-valence Pople's basis set for all the atomic species. The tetramers containing the PT moiety were optimized as an alternating copolymer of formula H–(–A–B–)_4_–H considering the regiochemical alternation between successive PT groups. For the sake of simplicity and to speed up the calculations, the alkyl chains were substituted with methyl groups in all investigated tetramers, a licit procedure as recently shown in the literature.^29^ The same level of theory was used for all the structural optimizations in gas-phase when we introduced the three different LAs to form the LAB adducts with the tetramer PFPT and PCPDTPT. We also checked the influence of the OT range separation parameter *ω* on the resulting optimized structures.^30^ Using a RSH functional often comes along with a non-empirical tuning of *ω*. In fact, for each specific N-electron system, an optimal value of *ω* can be found by enforcing the exchange-correlation functional to obey the DFT version of Koopman's theorem by aligning the negative energy of the HOMO with the gas-phase vertical IP. In practice, one computes the total energy difference between the N-electron and the (N-1)-electron system and tries to minimize the overall error by minimizing the following target function:

1 *J*^2^(*ω*)=(*ε*_HOMO_(*N*,*ω*) + IP(*N*,*ω*))^2^

In addition, for a better description of the fundamental gap, the gas-phase vertical EA of the N-electron system can be represented by the vertical IP of the (N+1)-electron system, barring relaxation effects. In order to perform a gap tuning procedure,^31–36^ the modified target function to minimize is the following:2
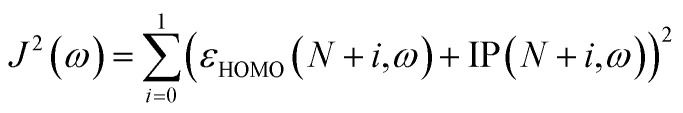


By doing that, the difference between the HOMO and LUMO energies of the N-electron systems in OT-RSH functionals provides a good approximation to the fundamental gap, that is the difference between IP and EA. In tuning the *ω* value, we resorted to a polarizable continuum model^37^ (PCM) using a screening dielectric constant of *ε* = 5.0, with the role of solvent polarity being addressed elsewhere.^29^ With this caveat, from now on, we will refer to the highest occupied molecular orbital (HOMO) negative energy as the vertical ionization potential (IP) of the molecule and to the lowest unoccupied molecular orbital (LUMO) negative energy as its vertical electron affinity (EA). For the neat PFPT and PCPDTPT tetramer and their relatives LAB adducts, the absorption spectra were computed with full TD-DFT calculations and a ground-state population analysis was performed by means of the Charge Model 5 (CM5),^38^ at the OT-RSH + PCM level of theory.

In order to identify the most likely protonation site by mimicking the protonation mediated by a Brønsted acid of the PCPDTBT tetramer, we modelled in a first place a CPDT-BT-CPDT unit (see sketch in Table 3). The pristine and protonated model moieties were tightly optimized in gas-phase at the *ω*B97X-D/6-31G(d,p) level of theory. Proton affinity (P(A)) is defined as the negative of the protonation reaction enthalpy at room temperature (*T* = 298 K):

3 *P*(*A*)=−ΔZPE − Δ*H*^0^_elec_ + 5/2*RT*

where ΔZPE is the corrected zero-point vibrational energy (ZPE) of the normal modes, Δ*H*^0^_elec_ is the variation in the electronic enthalpy going from the pristine to the protonated model moiety and *R* is the ideal gas constant. Then, in order to evaluate the thermodynamic properties of all the reactions presented below, each molecule was tightly optimized at the *ω*B97X-D/6-31G(d,p) level of theory in conjunction with PCM and *ε* = 5.0. The 3N − 6 frequencies of the vibrational normal modes (all checked to be positive) were computed and scaled by 0.949 in order to correct for anharmonicity effects.^39^ In a given reaction, the Gibbs free energy difference Δ*G*^0^ reads:

4 Δ*G*^0^(*T*) = Δ*H*^0^(*T*) − *T*Δ*S*^0^(*T*)

where Δ*H*^0^ is the enthalpy and Δ*S*^0^ is the entropy, both *T*-dependent. Moreover, each contribution can be decomposed in an electronic and a vibrational term (neglecting the rotational and translational ones, as they are not expected to contribute significantly), so that:

5 Δ*H*^0^(*T*) = Δ*H*^0^_elec_ + Δ*H*^0^_vib_(*T*)

6 Δ*S*^0^(*T*) = Δ*S*^0^_elec_ + Δ*S*^0^_vib_(*T*)

Within the harmonic approximation, the vibrational enthalpy *H*^0^_vib_(*T*) and the vibrational entropy *S*^0^_vib_(*T*) can be computed as:7
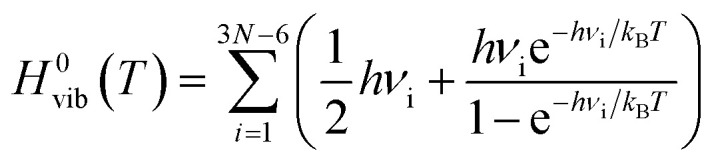
8
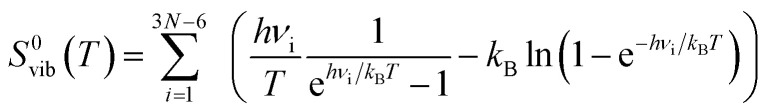
where *ν*_i_ is the frequency of the *i*-th normal mode, *h* is the Planck constant, *k*_B_ is the Boltzmann constant and both the sums run over the 3N − 6 normal modes. The electronic enthalpy *H*^0^_elec_ is directly computed at the DFT level, while the electronic entropy *S*^0^_elec_ can be estimated as:

9 *S*^0^_elec_ = *R* ln(2*S* + 1)

where *S* is the spin multiplicity. Here we present reactions at room temperature that involve neutral (*S* = 0) and radical (*S* = 1/2) species: thus, only the latter have an electronic entropic contribution. In each investigated reaction, its Δ*G*^0^ was computed as an energy difference between the products and the reactants, by calculating the enthalpic and entropic contribution of each species separately. DFT and TD-DFT calculations were performed using the GAUSSIAN16 package,^40^ while the calculations of the *g*-tensor values of the radical species presented in this work (see ESI and Fig. S10‡) were carried out resorting to the ORCA software^41^ at the DFT *ω*B97X-D/def2-TZVP level of theory, as recently done by some of us.^42^

## Results and discussion

The optimized pristine PFPT oligomer shows a rather twisted structure. Due to the steric repulsion experienced by the nearest hydrogen atoms in the fluorene group and the –CH side of the PT moiety (see Fig. S1 and Table S1 in ESI‡), the dihedral angles between these two groups are 39°, while the lower steric bulk on the N-bearing side of the PT results in a smaller PT/fluorene dihedral angles of 17–19°. Irrespective of its nature, the addition of one LA borane molecule with the boron atom in front of the pyridyl nitrogen in the PT group increases the dihedral angle up to 49–52°, while the other dihedrals further away from the LA remain unaltered. Gas-phase LAB adduct binding energies were estimated for the three LAs as total energy differences between the adduct coordinated with a LA and the sum of the isolated neat oligomer and LA molecule. The calculated binding energies prove the higher affinity of BBr_3_ (−29.5 kcal mol^−1^), followed by BCF and BF_3_ (−22.7 kcal mol^−1^ and −21.3 kcal mol^−1^, respectively), in line with previous theoretical and experimental works.^43,44^

**Fig. 2:**
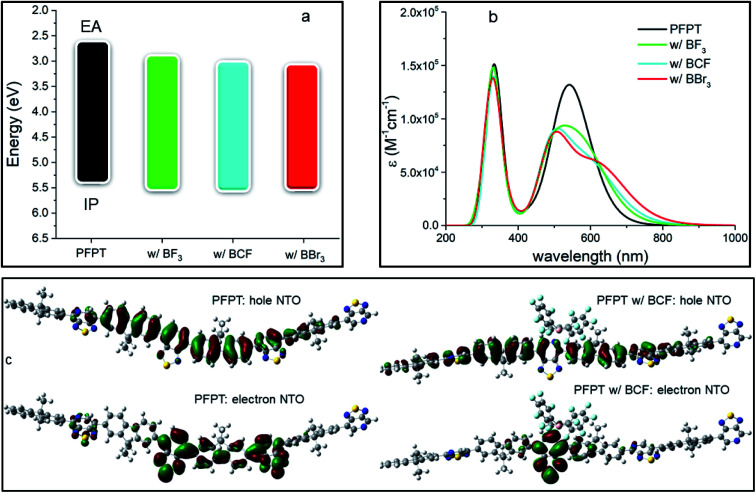
(a) Energetic diagram showing IP and EA (in eV), (b) calculated TD-DFT optical absorption spectra (in nm) for the different species at 0.25 LA molar equivalents and (c) lowest electronic excitation NTOs of the neat PFPT tetramer and the adduct with BCF. In panel (b) absorption spectra were convoluted with a full width half maximum of 0.2 eV and the molar absorption coefficient ε is reported on the *y*-axis.

The vertical IP and EA values of the neat PFPT oligomer and the corresponding adducts are reported in Table 1 (see also Fig. 2a). A clear stabilization of the charge-transport energy levels is observed in presence of LAs, *i.e.*, both the IP and EA of the LAB adducts are increased. These changes are asymmetric, with a larger impact on EA than IP, resulting in a lowering of the transport gap, *E*_gap_. In the case of BCF, the IP increases by 0.14 eV and the EA by 0.39 eV, for an overall reduction in *E*_gap_ of 0.25 eV. The changes in IP and EA are mostly driven by the partial ground-state CT taking place from the PT group to the LA, with changes across the series BBr_3_, BCF and BF_3_ also reflecting various degrees of hybridization of the unoccupied electronic levels (see ESI and Fig. S2‡). The predicted ∼0.1 eV change in IP upon complexation with BCF agrees with ultraviolet photoemission data.^13^

**Table 1:** Calculated IP, EA and transport gap *E*_gap_ (in eV) for the neat PFPT tetramer and for the different Lewis acid-base (LAB) adducts. Excitation wavelength (in nm), energy (in eV) and oscillator strength (*f*) of the lowest electronic transition S_0_–S_1_ are also reported

	IP	EA	*E*_gap_	*E*(S_0_–S_1_)	*f*(S_0_–S_1_)
PFPT	5.43	2.62	2.81	546/2.27	2.23
w/BF_3_	5.55	2.89	2.66	586/2.12	1.16
w/BCF	5.57	3.01	2.56	607/2.04	0.92
w/BBr_3_	5.57	3.11	2.46	628/1.97	0.92

TD-DFT calculations (Fig. 2b, Table 1) indicate the emergence of a new, red-shifted, optical absorption band upon complexation.^45^ As detailed below, the additional optical feature at wavelengths above 600 nm directly reflects the section of the polymer backbone interacting with the LA, with regions spatially away from the contact points contributing to the feature that is seen at ∼520–550 nm, slightly blue-shifted from that of the neat oligomer. We observe the largest red-shift of the lowest electronic excitation for BBr_3_ (0.30 eV), followed by BCF (0.23 eV) and BF_3_ (0.15 eV). The predicted red-shift (by 0.23 eV) of the lowest electronic transition is in excellent agreement with experimental optical absorption at 1 molar equivalent and above of BCF, showing a ∼0.3 eV red-shift of the maximum absorption peak in both film and solution.^13^ Natural transition orbitals (NTOs) pertaining to the lowest electronic excitation of the neat oligomer and the adduct with BCF are reported in Fig. 2c. In the neat PFPT oligomer, the hole density is delocalized over the entire molecular backbone, but the electron density has larger weights on the PT electron-accepting units (with dominant contributions on the two inner rings), consistent with the lowest excited state having significant intramolecular CT character. When BCF binds a pyridyl nitrogen on the PT group, the hole density distribution remains essentially unaltered (despite the slight increase in IP relative to the pristine oligomer), and the electron density is now fully confined to the PT moiety that is in direct interaction with the LA (as this PT unit is now electron poorer and has higher EA). The lowest electronic excitation NTOs of the adduct with BF_3_ and BBr_3_ are shown in Fig. S3 in ESI.‡ In order to assess the influence of polymer chain length and its potential impact on the nature of the optical excitations,^46^ we also modelled a neat PFPT octamer and its LAB adduct with BCF (see Table S2 and Fig. S4 in ESI‡). By doubling the molecular length, we note that *E*_gap_ is only slightly reduced (by ∼0.1 eV), mainly due to a destabilization of the IP. Irrespective of the conjugation length, the lowest electronic transition of the LAB adduct is red-shifted by 0.20 eV compared to the neat polymer chain.

**Fig. 3:**
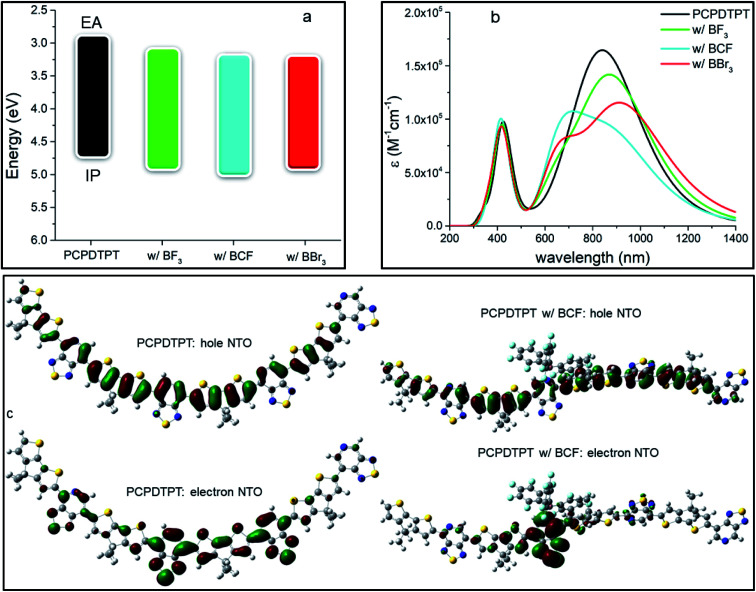
(a) Energetic diagram showing IP and EA (in eV), (b) calculated TD-DFT optical absorption spectra (in nm) for the different species at 0.25 LA molar equivalents and (c) lowest electronic excitation NTOs of the neat PCPDTPT tetramer and the adduct with BCF. In panel (b) absorption spectra were convoluted with a full width half maximum of 0.2 eV and the molar absorption coefficient ε is reported on the *y*-axis.

We performed the same analysis for another donor–acceptor oligomer, PCPDTPT, differing from PFPT by the nature of the electron-donating units (see Fig. S5 and Table S3 in ESI‡). In contrast to PFPT, the PCPDTPT oligomer has a perfectly planar backbone with all dihedral angles equal to 0° in the pristine form. However, the addition of a LA molecule dramatically distorts the structure of the oligomer because of steric effects: the bulkier the LA, the higher the degree of distortion. In particular, the dihedral angle between the LA-bound side of the PT and the CPDT moiety reaches 112° (almost orthogonal orientation) in the adduct formed with BCF, 46° with BBr_3_ and 39° with BF_3_. We stress that these substantial changes in the conformation of the molecular backbone are expected to strongly perturb the optical properties of the LAB adduct, as a result of the reduced π-conjugation. A similar effect was also observed by Schier *et al.*^47^ for a quarterthiophene (4T) doped by BCF, with the presence of the LA interacting with the oligomer inducing substantial structural distortions. The calculated IP and EA values of the neat PCPDTPT and its respective LAB adducts, reported in Table 2 and Fig. 3a, show that, upon binding, there is an effective decrease in *E*_gap_.

**Table 2:** Calculated IP, EA and *E*_gap_ (in eV) for the neat PCPDTPT tetramer and for the different LAB adducts. Excitation wavelength (in nm), energy (in eV) and oscillator strength (*f*) of the lowest electronic transition S_0_–S_1_ are also reported

	IP	EA	*E*_gap_	*E*(S_0_–S_1_)	*f*(S_0_–S_1_)
PCPDTPT	4.73	2.89	1.84	864/1.43	2.68
w/BF_3_	4.88	3.08	1.80	892/1.39	2.41
w/BCF	4.96	3.17	1.79	910/1.36	1.26
w/BBr_3_	4.93	3.23	1.70	940/1.32	1.92

However, this effect is far less pronounced than for the PFPT oligomer, with the largest lowering of *E*_gap_ being 0.14 eV in the case of BBr_3_ (*versus* 0.35 eV for PFPT:BBr_3_). As in the PFPT case, the IP, EA and *E*_gap_ values are dictated by a partial ground-state CT and orbital hybridization in the LUMO of the adduct (see ESI and Fig. S6‡). We attribute the reduced spectral change to a competition to the opposing effects exerted by electronic CT and hybridization (which tend to reduce the gap) and conformational distortions away from planarity (which tend to increase the gap).

TD-DFT optical absorption spectra in Fig. 3b (see also Table 2) show that the formation of the LAB adduct is accompanied by the appearance of a new, red-shifted, optical transition fingerprint, as in the PFPT case. The largest red-shift is predicted for BBr_3_ (0.11 eV), followed by BCF (0.07 eV) and BF_3_ (0.04 eV), following the trend of the calculated *E*_gap_ values and similar to what reported above for PFPT. We also note that optical absorption measurements on PCPDTPT:BCF thin films point to a larger spectral shift (reaching almost 0.4 eV)^16^ than predicted, a discrepancy that could arise from conformational restraints in the solid-state (see Fig. S7 and Table S4 in ESI‡). The first excitation NTOs of the adduct with BF_3_ and BBr_3_ are shown in Fig. S8 in ESI.‡

**Scheme 1:**
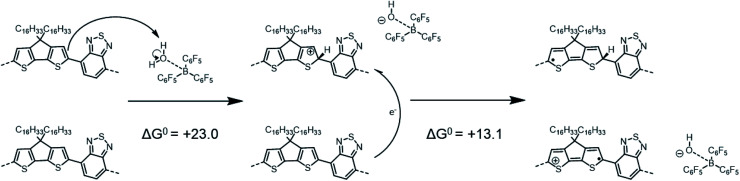
Reaction mechanism similar to that proposed by Yurash *et al.*, involving a protonation followed by an electron-transfer reaction (this mechanism differs from that in ref. 16 in the position of the protonated site, see below). Calculations reported here yield Δ*G*^0^ = +23.0 kcal mol^−1^ (or +1.00 eV) for the protonation and Δ*G*^0^ = +13.1 kcal mol^−1^ (or +0.57 eV) for the electron transfer. For the sake of simplicity, the distinct structures are shown for single tetramer repeat unit, while we acknowledge that both spin and charge will be delocalized over multiple repeat units to varying extents.

In contrast to the previous two tetramers that were investigated, PCPDTBT does not undergo any binding reaction with LAs,^16^ as the benzothiadiazole (BT) moiety lacks a pyridyl nitrogen able to share an electron lone pair with the empty boron p-orbital of the LA. Instead, adding BCF to a PCPDTBT based film leads to an increase in electrical conductivity and to the formation of positive polarons, *i.e.*, molecular p-doping.^16,24,48^ As in the mechanism proposed by Doerrer and Green for oxidation of metallocenes,^14^ Yurash *et al.* suggested that the first step of this p-doping was the protonation by the highly Brønsted acidic complex BCF(OH_2_) of the CPDT moiety of the polymer backbone.^16^ They further proposed that protonation would increase the EA sufficiently that a nearby neutral chain segment would be able to transfer an electron to the (positively charged) protonated segment (with the segments belonging either to the same or different physical polymer chains, if the process is intrachain or interchain, respectively). This mechanism results in the formation of two radical species: a neutral, “protonated radical” and a radical cation, as shown in Scheme 1:

The optimized PCPDTBT structure in PCM yields a slightly twisted backbone, with all the dihedral angles of about 20° (see Fig. S9 and Table S5 in ESI‡). In an attempt to identify the most likely protonation site along the polymer backbone, we performed *P*(*A*) calculations. The results reported in Table 3 show that (in contrast to ref. 16 in which position 3 was assumed to be protonated) position 1 (an α-carbon atom) in the CPDT moiety is the most favorable site to be protonated, followed by position 3 (a β-carbon atom) and 2. As a result, the Δ*H*^0^_elec_ penalty for the protonation step is significantly overestimated in the modeling work by Yurash *et al.* compared to the value reported here (+40.4 kcal mol^−1^ in ref. 16*versus* +22.9 kcal mol^−1^ here). The addition of one proton (or hydrogen atom) to position 1 on the CPDT group dramatically affects the polymer backbone planarity since it breaks the π-conjugation by introducing sp^3^ carbon atoms and the oligomer becomes quite twisted. By computing the thermodynamic properties of all the species (*i.e.*, proposed reactants, intermediates and products) involved in the above reactions, our calculations show that both the protonation and the one electron-transfer processes are substantially endergonic, with Δ*G*^0^ values of +23.0 and +13.1 kcal mol^−1^, respectively (see Scheme 1), implying a total Δ*G*^0^ of +36.1 kcal mol^−1^ (or +1.57 eV), thus suggesting the overall reaction to be very unlikely.

**Table 3:** Corrected zero-point vibrational energies (ΔZPEs), electronic enthalpy variations (Δ*H*^0^_elec_) going from the pristine model CPDT-BT-CPDT moiety to the protonated one. Proton affinities (*P*(*A*)) calculations for different protonating sites, as highlighted in the sketch below, were performed in gas-phase. All values in the table are expressed in kcal mol^−1^

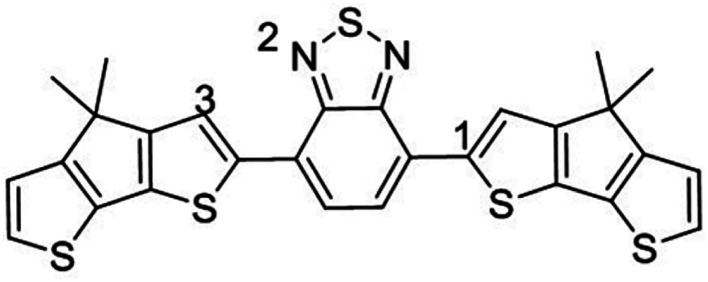			
	*Δ*ZPE	Δ*H*^0^_elec_	*P*(*A*)
1	7.22	−237.70	231.96
2	7.06	−229.51	223.94
3	6.67	−232.95	227.76

**Scheme 2:**
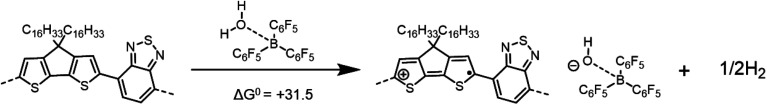
Overall p-doping reaction proposed by Arvind *et al.* for P3HT as applied to the case of PCPDTBT. DFT calculations indicate a total Δ*G*^0^ = +31.5 kcal mol^−1^ (or +1.36 eV) for this reaction.

**Scheme 3:**
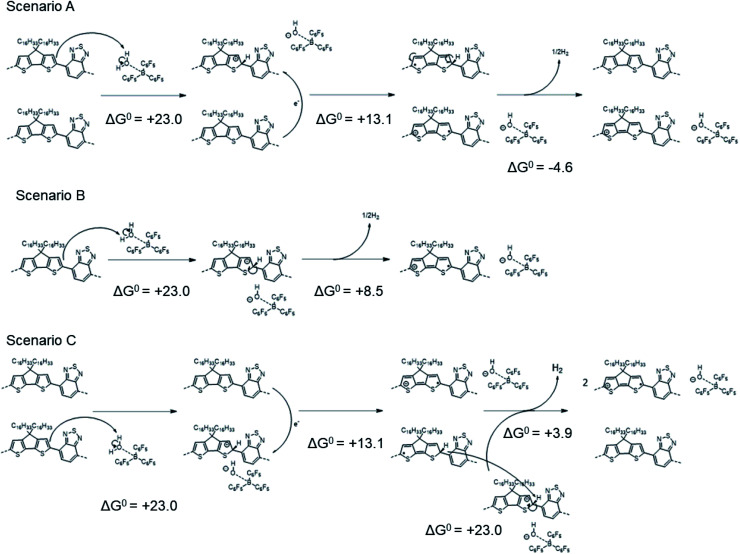
Different mechanisms that might afford the same overall reaction as that shown in Scheme 2.

In a recent study by Arvind *et al.*^24^ on P3HT, EPR measurements performed on BCF-doped samples revealed the formation of free radical cations on the polymer backbone, yet showing no indication for the presence of another radical species (*i.e.*, associated with the “protonated radical”). If BCF doping of PCPDTBT proceeds in analogous fashion to that proposed for the BCF-induced doping of P3HT by Arvind *et al.* the overall reaction would be that shown in Scheme 2:

The computed Δ*G*^0^ value for the overall reaction is +31.5 kcal mol^−1^, smaller than that for Scheme 1, but still highly endergonic. As shown in Scheme 3, several possible pathways might lead to the same overall reaction as that shown in Scheme 2:

In scenario A, the protonation step is followed by electron transfer (as in Scheme 1), but here two neutral “protonated radicals” subsequently react to eliminate H_2_ to regenerate two neutral closed-shell polymers (shown for one such radical affording half a molecule of H_2_), contributing a negative (exergonic) Δ*G*^0^ = −4.6 kcal mol^−1^ (or −0.20 eV). Scenario B is a variant of scenario A where H_2_ is eliminated from two protonated cationic polymers, contributing with a Δ*G*^0^ = +8.5 (13.1–4.6) kcal mol^−1^ (or +0.37 eV). Finally, scenario C is a combination of scenarios A and B, leading, as expected, to a twofold increase in the total Δ*G*^0^ = +63.0 (2 × 31.5) kcal mol^−1^. We note that reactions of the type shown in Schemes 2 and 3 (and the similar overall reactions involving larger counter-ions that are discussed in the following section) are apparently at odds with the CW ENDOR results of ref. 16. However, although the structureless feature is consistent with that expected for the “protonated radical”, it could also in principle arise from dynamic effects leading to loss of the structure expected for the polaron signal, or even from other radicals formed through side reactions. We also reckon that, as observed elsewhere in the literature,^24,49–51^ the polymer conjugation length plays a paramount role in the context of molecular doping, since different mechanisms might occur depending on the extension of the polymer backbone. To address this point, Table S6 in ESI‡ reports the computed Δ*H*^0^_elec_ values pertaining to Scheme 1 and Scheme 2, using either a PCPDTBT tetramer or an octamer as representative model. The computed Δ*H*^0^_elec_ values are found to be comparable, which comforts our choice of tetramer models as providing a good trade-off between accuracy and computational cost.

**Scheme 4:**
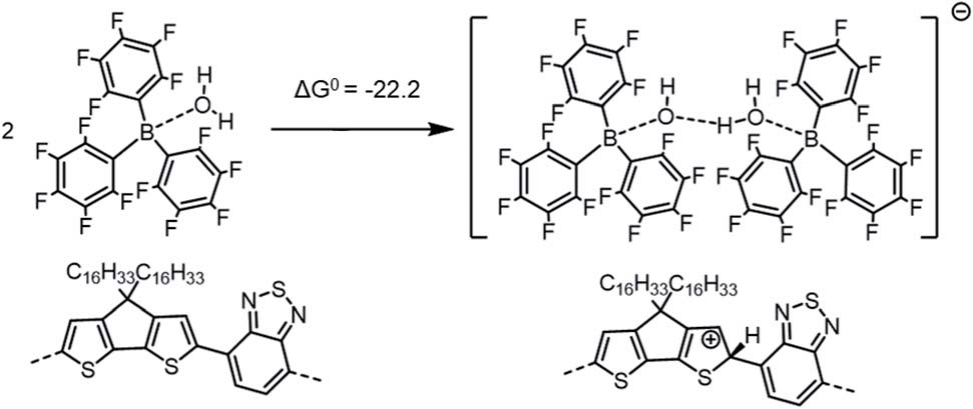
Formation of the [BCF(OH)(OH_2_)BCF]^−^ anion and protonation of the neat PCPDTBT tetramer, which in this case yields a negative (exergonic) Δ*G*^0^ = −22.2 kcal mol^−1^ (or −0.96 eV).

**Scheme 5:**
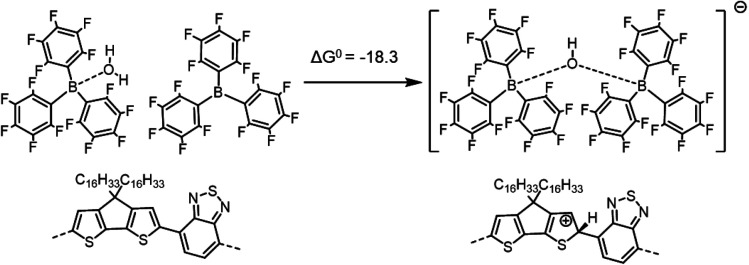
Formation of the [BCF(OH)BCF]^−^ anion and protonation of the neat PCPDTBT tetramer, yielding a negative (exergonic) Δ*G*^0^ = −18.3 kcal mol^−1^ (or −0.79 eV).

Neither the overall reactions of Scheme 1 nor Scheme 2 appear likely to represent the mechanism responsible for the formation of excess charge carriers in PCPDTBT upon LA doping, since the overall reactions are highly endergonic, with a particularly high energy penalty being associated with the protonation of the pristine polymer chains by BCF(OH_2_) complex with concomitant formation of [BCF(OH)]^−^. However, in the previous work on metallocene oxidation by BCF(OH_2_),^14^ [BCF(OH)]^−^ was not observed, but rather [BCF(OH)(OH_2_)BCF]^−^ (Scheme 4), in which [BCF(OH)]^−^ is hydrogen bonded to another BCF(OH_2_) complex, and [BCF(OH)BCF]^−^ (Scheme 5), where [BCF(OH)]^−^ coordinates a BCF molecule. We reconsidered, therefore, Scheme 2 based on that proposed by Arvind *et al.* for P3HT, but now forming anions containing two BCF units of the two types observed by Doerrer and Green:

If we consider the protonation reaction as forming the “four-body” [BCF(OH)(OH_2_)BCF]^−^ anion of Scheme 4, the highly endergonic (Δ*G*^0^ = +23.0 kcal mol^−1^) protonation reaction found when [BCF(OH)]^−^ is formed now becomes highly exergonic (Δ*G*^0^ = −22.2 kcal mol^−1^). Consequently, the overall Δ*G*^0^ for the reaction presented in Scheme 1 and that for a H_2_-forming reaction in Scheme 2 is now negative: −9.0 kcal mol^−1^ (or −0.39 eV) for the former and −13.7 kcal mol^−1^ (or −0.59 eV) for the latter. Moreover, none of the proposed steps after protonation is prohibitively endergonic, and thus may be kinetically feasible, while irreversible loss of gaseous H_2_ can drive the doping reaction to the right. The greater exergonicity arises from stabilization of the counterion by delocalization of excess negative charge over two BCF molecules *via* the [OH–H_2_O]^−^ stabilizing bridge (see Fig. S11 in ESI‡). A similar, though smaller in magnitude, delocalization of the excess negative charge occurs when [BCF(OH)BCF]^−^ is formed (see Fig. S12 in ESI‡), which results in a slightly less, but still, favorable protonation reaction in Scheme 5, with Δ*G*^0^ = −18.3 kcal mol^−1^. This means also that the intermediate neutral [BCF(OH_2_)BCF] complex (not shown in Scheme 5) is more stable than BCF:OH_2_ and BCF separately. The global Δ*G*^0^ in that case amounts to −5.1 kcal mol^−1^ (or −0.22 eV) for the reaction in Scheme 1 and −9.7 kcal mol^−1^ (or −0.42 eV) for the reaction in Scheme 2. Finally, an alternative reaction pathway regarding the [BCF(OH)BCF]^−^ formation is reported in Fig. S13 in ESI,‡ when two BCF(OH_2_) complexes react together and an H_2_O molecule is eliminated, yielding a slightly negative Δ*G*^0^ of −0.2 kcal mol^−1^ (or −9 meV, within *k*_B_*T*). Thus, when all the BCF molecules are complexed by H_2_O, the reaction presented in Scheme 4 is the most exergonic. On the other hand, the new protonation reaction in Fig. S13‡ could occur if water is somehow removed.

## Conclusions

We modelled the interactions between three boron-based LAs and different semiconducting π-conjugated polymers, performing detailed quantum-chemical calculations of the structural, energetics and optical signatures for ground-state LAB adducts between LAs and either PFPT or PCPDTPT. Our calculations demonstrate that the observed red-shifted optical absorption in the adducts results from a complex interplay between hybridization, partial CT and changes in the polymer conformation. In assessing the potential of BCF to induce molecular doping in PCPDTBT based on calculated Gibbs free energies of different proposed reactions, we came to the conclusions that both the overall processes proposed by Yurash *et al.*^16^ and by Arvind *et al.*^24^ are highly endergonic, mostly because of the thermodynamically unfavourable protonation by BCF(OH_2_). Reconciling theory with experiment requires considering complexation of the [BCF(OH)]^−^ with another BCF or BCF(OH_2_) moiety to form more stable anions of the stoichiometry and structure observed crystallographically by Doerrer and Green;^14^ these offer a dramatic reduction in the Δ*G*^0^ penalty for forming the protonated intermediates. We propose that this is followed by moderately endergonic reactions resulting in the elimination of H_2_ (as also suggested for the case of metallocene oxidation), either directly from two protonated cationic segments of polymer chains, from “protonated radicals” formed by electron transfer between neutral and protonated cationic segments, or from a protonated cation and a protonated radical (Scheme 3), hence explaining why a single spin-carrying species is observed in EPR measurements. Overall, our calculations highlight the necessity of H_2_ loss for the overall feasibility of the reaction, and most importantly, the key role played by the formation of diboron-containing bridged anions in the doping mechanism. Those bridged anions were known, as was the monomeric [BCF(OH)]^−^, but the energetic benefits of bridged anion formation, and therefore its effect on overall reaction feasibility, had not been recognized and certainly not quantified, neither in ref. 16 nor in other works dealing with the doping of π-conjugated polymers with LAs.

This is the likely mechanism prevailing at dopant concentrations large enough that BCF dopants can encounter and form complex anions derived from two BCF moieties. In addition, at low dopant concentration and if the dopant is rigorously water-free, it is also possible that highly hygroscopic BCF molecules could free hole carriers from trapping sites associated with water and/or water–oxygen complexes,^52,53^ rather than create excess charges through a conventional doping mechanism. Additional experimental and theoretical work is needed to confirm or reject this hypothesis, as well as to unravel the exact nature of the BCF(OH_2_) adducts present in doping solutions and the anions present in doped solids. However, this is likely to be very challenging as, even in solution, ^1^H and ^19^F NMR spectroscopies are unable to reliably distinguish between BCF(OH_2_)_*n*_ complexes with different *n*,^54^ while neither the ^11^B nor ^19^F NMR spectra of [BCF(OH)BCF]^−^ differ significantly from that of [BCF(OH)(OH_2_)BCF]^−^ in solution.^22^ Finally we note that the non-straightforward doping nature of the BCF-induced doping process potentially complicates predictions regarding its applicability to other semiconductors. Although variations of the thermodynamic feasibility of the proposed overall p-doping reaction (Scheme 2, but with a complex counterion) for different semiconductors will depend only on the IP of the semiconductor, the kinetic feasibility is expected to depend critically on the ability to protonate the semiconductor. Moreover, different mechanisms may be operative for different semiconductors, for example, if they form substantially more stable “protonated radicals” than PCPDTBT. Finally, the use of BCF as a p-dopant relies on adventitious water and to obtain reproducible doping levels it is likely desirable to use a well-defined and intentionally synthesized BCF(OH_2_) complex. However, in the presence of additional adventitious water the Brønsted acidity (and thus oxidant strength) of BCF(OH_2_) is likely decreased. In addition, BCF(OH_2_) decomposes to (C_6_F_5_)_2_BOH and C_6_F_5_H on heating,^55^ potentially leading to an ill-defined mixture of species in doping solutions or doped films. It will be useful to carry out further work to identify other Brønsted acids that may be used as effective dopants and that avoid some of these drawbacks.

## Author contributions

P. S. M., G. L. and D. B. conceived the work. P. S. M. and G. L. performed the quantum chemical calculations. G. L. and D. B. wrote the manuscript with valuable inputs from all the authors.

## Conflicts of interest

The authors declare no competing financial interest.

## Supplementary Material

SC-012-D1SC01268A-s001

## References

[cit1] Salzmann I., Heimel G., Oehzelt M., Winkler S., Koch N. (2016). Acc. Chem. Res..

[cit2] Mityashin A., Olivier Y., Van Regemorter T., Rolin C., Verlaak S., Martinelli N. G., Beljonne D., Cornil J., Genoe J., Heremans P. (2012). Adv. Mater..

[cit3] Méndez H., Heimel G., Opitz A., Sauer K., Barkowski P., Oehzelt M., Soeda J., Okamoto T., Takeya J., Arlin J.-B., Balandier J.-Y., Geerts Y., Koch N., Salzmann I. (2013). Angew. Chem., Int. Ed..

[cit4] Lüssem B., Riede M., Leo K. (2013). Phys. Status Solidi.

[cit5] Li J., Duchemin I., Roscioni O. M., Friederich P., Anderson M., Da Como E., Kociok-Köhn G., Wenzel W., Zannoni C., Beljonne D., Blase X., D'Avino G. (2019). Mater. Horiz..

[cit6] Privitera A., Londi G., Riede M., D'Avino G., Beljonne D. (2020). Adv. Funct. Mater..

[cit7] Bridges C. R., Baumgartner T. (2020). J. Phys. Org. Chem..

[cit8] Welch G. C., Coffin R., Peet J., Bazan G. C. (2009). J. Am. Chem. Soc..

[cit9] Zalar P., Henson Z. B., Welch G. C., Bazan G. C., Nguyen T. Q. (2012). Angew. Chem., Int. Ed..

[cit10] Zalar P., Kuik M., Henson Z. B., Woellner C., Zhang Y., Sharenko A., Bazan G. C., Nguyen T.-Q. (2014). Adv. Mater..

[cit11] Han Y., Barnes G., Lin Y.-H., Martin J., Al-Hashimi M., AlQaradawi S. Y., Anthopoulos T. D., Heeney M. (2016). Chem. Mater..

[cit12] Panidi J., Paterson A. F., Khim D., Fei Z., Han Y., Tsetseris L., Vourlias G., Patsalas P. A., Heeney M., Anthopoulos T. D. (2018). Adv. Sci..

[cit13] Yurash B., Leifert D., Reddy G. N. M., Cao D. X., Biberger S., V Brus V., Seifrid M., Santiago P. J., Köhler A., Chmelka B. F., Bazan G. C., Nguyen T.-Q. (2019). Chem. Mater..

[cit14] Doerrer L. H., Green M. L. H. (1999). J. Chem. Soc., Dalton Trans..

[cit15] Danopoulos A. A., Galsworthy J. R., Green M. L. H., Doerrer L. H., Cafferkey S., Hursthouse M. B. (1998). Chem. Commun..

[cit16] Yurash B., Cao D. X., Brus V. V., Leifert D., Wang M., Dixon A., Seifrid M., Mansour A. E., Lungwitz D., Liu T., Santiago P. J., Graham K. R., Koch N., Bazan G. C., Nguyen T. Q. (2019). Nat. Mater..

[cit17] Lawrence E. J., Oganesyan V. S., Wildgoose G. G., Ashley A. E. (2013). Dalton Trans..

[cit18] Pingel P., Arvind M., Kölln L., Steyrleuthner R., Kraffert F., Behrends J., Janietz S., Neher D. (2016). Adv. Electron. Mater..

[cit19] Mohapatra S. K., Zhang Y., Sandhu B., Fonari M. S., V Timofeeva T., Marder S. R., Barlow S. (2016). Polyhedron.

[cit20] Aubry T. J., Axtell J. C., Basile V. M., Winchell K. J., Lindemuth J. R., Porter T. M., Liu J.-Y., Alexandrova A. N., Kubiak C. P., Tolbert S. H., Spokoyny A. M., Schwartz B. J. (2019). Adv. Mater..

[cit21] Yan H., Chen J., Zhou K., Tang Y., Meng X., Xu X., Ma W. (2018). Adv. Energy Mater..

[cit22] Yan H., Tang Y., Sui X., Liu Y., Gao B., Liu X., Liu S. F., Hou J., Ma W. (2019). ACS Energy Lett..

[cit23] Yan H., Tang Y., Meng X., Xiao T., Lu G., Ma W. (2019). ACS Appl. Mater. Interfaces.

[cit24] Arvind M., Tait C. E., Guerrini M., Krumland J., Valencia A. M., Cocchi C., Mansour A. E., Koch N., Barlow S., Marder S. R., Behrends J., Neher D. (2020). J. Phys. Chem. B.

[cit25] Abate A., Hollman D. J., Teuscher J., Pathak S., Avolio R., D'Errico G., Vitiello G., Fantacci S., Snaith H. J. (2013). J. Am. Chem. Soc..

[cit26] Körzdörfer T., Brédas J. L. (2014). Acc. Chem. Res..

[cit27] Kronik L., Kümmel S. (2018). Adv. Mater..

[cit28] Lin Y. S., De Li G., Mao S. P., Da Chai J. (2013). J. Chem. Theory Comput..

[cit29] Krumland J., Valencia A. M., Cocchi C. (2021). Phys. Chem. Chem. Phys..

[cit30] Marqués P. S., Andrés Castán J. M., Raul B. A. L., Londi G., Ramirez I., Pshenichnikov M. S., Beljonne D., Walzer K., Blais M., Allain M., Cabanetos C., Blanchard P. (2020). Chem.–Eur. J..

[cit31] Stein T., Kronik L., Baer R. (2009). J. Am. Chem. Soc..

[cit32] Stein T., Kronik L., Baer R. (2009). J. Chem. Phys..

[cit33] Refaely-Abramson S., Baer R., Kronik L. (2011). Phys. Rev. B: Condens. Matter Mater. Phys..

[cit34] Refaely-Abramson S., Sharifzadeh S., Govind N., Autschbach J., Neaton J. B., Baer R., Kronik L. (2012). Phys. Rev. Lett..

[cit35] Kronik L., Stein T., Refaely-Abramson S., Baer R. (2012). J. Chem. Theory Comput..

[cit36] Refaely-Abramson S., Sharifzadeh S., Jain M., Baer R., Neaton J. B., Kronik L. (2013). Phys. Rev. B: Condens. Matter Mater. Phys..

[cit37] Tomasi J., Mennucci B., Cammi R. (2005). Chem. Rev..

[cit38] Marenich A. V., Jerome S. V., Cramer C. J., Truhlar D. G. (2012). J. Chem. Theory Comput..

[cit39] NIST Computational Chemistry Comparison and Benchmark Database, http://cccbdb.nist.gov/

[cit40] Frisch M. J., Trucks G. W., Schlegel H. B., Scuseria G. E., Robb M. A., Cheeseman J. R., Scalmani G., Barone V., Petersson G. A., Nakatsuji H., Li X., Caricato M., Marenich A. V., Bloino J., Janesko B. G., Gomperts R., Mennucci B., Hratchian H. P., Ortiz J. V., Izmaylov A. F., Sonnenberg J. L., Williams-Young D., Ding F., Lipparini F., Egidi F., Goings J., Peng B., Petrone A., Henderson T., Ranasinghe D., Zakrzewski V. G., Gao J., Rega N., Zheng G., Liang W., Hada M., Ehara M., Toyota K., Fukuda R., Hasegawa J., Ishida M., Nakajima T., Honda Y., Kitao O., Nakai H., Vreven T., Throssell K., Montgomery Jr J. A., Peralta J. E., Ogliaro F., Bearpark M. J., Heyd J. J., Brothers E. N., Kudin K. N., Staroverov V. N., Keith T. A., Kobayashi R., Normand J., Raghavachari K., Rendell A. P., Burant J. C., Iyengar S. S., Tomasi J., Cossi M., Millam J. M., Klene M., Adamo C., Cammi R., Ochterski J. W., Martin R. L., Morokuma K., Farkas O., Foresman J. B. and Fox D. J., Gaussian 16, Revision A.03, Gaussian, Inc., Wallingford CT, 2016

[cit41] Neese F. (2012). Wiley Interdiscip. Rev.: Comput. Mol. Sci..

[cit42] Privitera A., Warren R., Londi G., Kaienburg P., Liu J., Sperlich A., Lauritzen A. E., Thimm O., Ardavan A., Beljonne D., Riede M. (2021). J. Mater. Chem. C.

[cit43] Jonas V., Frenking G., Reetz M. T. (1994). J. Am. Chem. Soc..

[cit44] Mo Y., Gao J. (2001). J. Phys. Chem. A.

[cit45] Phan H., Kelly T. J., Zhugayevych A., Bazan G. C., Nguyen T. Q., Jarvis E. A., Tretiak S. (2019). J. Phys. Chem. Lett..

[cit46] Valencia A. M., Cocchi C. (2019). J. Phys. Chem. C.

[cit47] Schier R., Valencia A. M., Cocchi C. (2020). J. Phys. Chem. C.

[cit48] Wegner B., Lungwitz D., Mansour A. E., Tait C. E., Tanaka N., Zhai T., Duhm S., Forster M., Behrends J., Shoji Y., Opitz A., Scherf U., List-Kratochvil E. J. W., Fukushima T., Koch N. (2020). Adv. Sci..

[cit49] Pingel P., Zhu L., Park K. S., Vogel J.-O., Janietz S., Kim E.-G., Rabe J. P., Brédas J.-L., Koch N. (2010). J. Phys. Chem. Lett..

[cit50] Méndez H., Heimel G., Winkler S., Frisch J., Opitz A., Sauer K., Wegner B., Oehzelt M., Röthel C., Duhm S., Többens D., Koch N., Salzmann I. (2015). Nat. Commun..

[cit51] Mansour A. E., Lungwitz D., Schultz T., Arvind M., Valencia A. M., Cocchi C., Opitz A., Neher D., Koch N. (2020). J. Mater. Chem. C.

[cit52] Nikolka M., Nasrallah I., Rose B., Ravva M. K., Broch K., Sadhanala A., Harkin D., Charmet J., Hurhangee M., Brown A., Illig S., Too P., Jongman J., McCulloch I., Brédas J.-L., Sirringhaus H. (2017). Nat. Mater..

[cit53] Nikolka M., Schweicher G., Armitage J., Nasrallah I., Jellett C., Guo Z., Hurhangee M., Sadhanala A., McCulloch I., Nielsen C. B., Sirringhaus H. (2018). Adv. Mater..

[cit54] Beringhelli T., Maggioni D., D'Alfonso G. (2001). Organometallics.

[cit55] Bradley D. C., Harding I. S., Keefe A. D., Motevalli M., Zheng D. H. (1996). J. Chem. Soc., Dalton Trans..

