# Supplementary material for: Understanding how Lewis acids dope organic semiconductors: a “complex” story
Source: Chem Sci. 2021 Apr 19;12(20):7012–22. doi: 10.1039/d1sc01268a (PMC8153436; doi:10.1039/d1sc01268a)
Supplement: SC-012-D1SC01268A-s001 [file SC-012-D1SC01268A-s001.pdf]

Electronic Supplementary Material (ESI) for Chemical Science.
This journal is © The Royal Society of Chemistry 2021

**Electronic Supporting Information for:**

# Understanding How Lewis Acids Dope Organic Semiconductors: A "Complex" Story

Pablo Simón Marqués[a], Giacomo Londi[b], Brett Yurash[c], Thuc-Quyen Nguyen[c], Stephen Barlow[d], Seth R. Marder[d] and David Beljonne[b*]

*a) Laboratoire MOLTECH-Anjou, UMR CNRS 6200, UNIV Angers, SFR MATRIX, 2 Bd Lavoisier, 49045 Angers Cedex, France*

*b) Laboratory for Chemistry of Novel Materials, University of Mons, Place du Parc, 20, 7000 Mons, Belgium*

*c) Center for Polymers and Organic Solids, Department of Chemistry & Biochemistry, University of California, Santa Barbara, California 93106, United States of America*

*d) Center for Organic Photonics and Electronics, School of Chemistry and Biochemistry, Georgia Institute of Technology, Atlanta, Georgia, 30332-0400, United States of America*

*email: david.beljonne@umons.ac.be

Contents

I. PFPT . . . . . . . . . 2
II. PCPDTPT . . . . . . . . 6
III. PCPDTBT . . . . . . . . 9
IV. BCF complexes with $H_2O$ . . . . . . 11

## I. PFPT

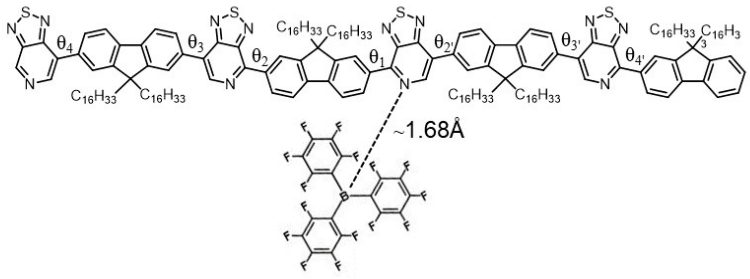


*Figure S1: Chemical structure of the PFPT tetramer bound to BCF.*

*Table S1: Dihedral angles of the neat PFPT tetramer and the different adducts.*

| | $\theta_1$ | $\theta_2$ | $\theta_{2'}$ | $\theta_3$ | $\theta_{3'}$ | $\theta_4$ | $\theta_{4'}$ |
|---|---|---|---|---|---|---|---|
| **Neat** | 17 | 19 | 39 | 39 | 39 | 39 | 19 |
| **w/ $BF_3$** | 49 | 19 | 39 | 39 | 39 | 39 | 19 |
| **w/ BCF** | 51 | 19 | 41 | 39 | 39 | 39 | 19 |
| **w/ $BBr_3$** | 52 | 19 | 37 | 39 | 39 | 39 | 19 |

We performed a ground-state CM5 charge population analysis for the neat PFPT oligomer and when bound to LAs. While the PT group in the neat form carries a slightly negative partial charge (-0.07|e|), it acts as a LB within the coordinated complex, bearing a positive partial charge of about +0.23|e|, irrespective of the LA. As expected, the LA withdraws electron density from the PT group (making it more electron-poor), carrying a negative partial charge of -0.40|e|. The sum of the net charges upon binding is negative, as there is also a charge-transfer (CT) contribution arising from adjacent regions in the oligomer. The ground-state CT character of the adducts does not explain the trend observed on the EA, $E_{gap}$, and the absorption peak in Table 1 (see main text) with the nature of the LA. We found instead instructive to quantify the contribution of the LA to the LUMO wave function in the adduct: $BF_3$ contributes much less (~0.5%) to the LUMO than BCF (~3.0%) or $BBr_3$ (~2.6%). There is thus some partial hybridization of the oligomer molecular orbitals with the boron atom of the LA; the variation of this hybridization with LA, combined with the partial ground-state CT, helps rationalize why, in comparison to the other two LAs, $BF_3$ shows the smallest change in the EA and, in turn, the smallest impact on the $E_{gap}$ of the adduct.

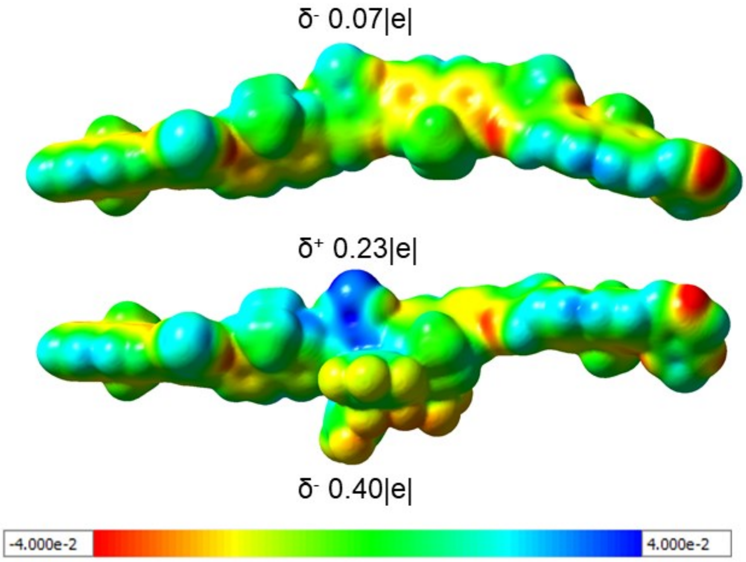


*Figure S2: CM5 charge distribution of the neat tetramer (top) and the adduct with BCF (bottom).*

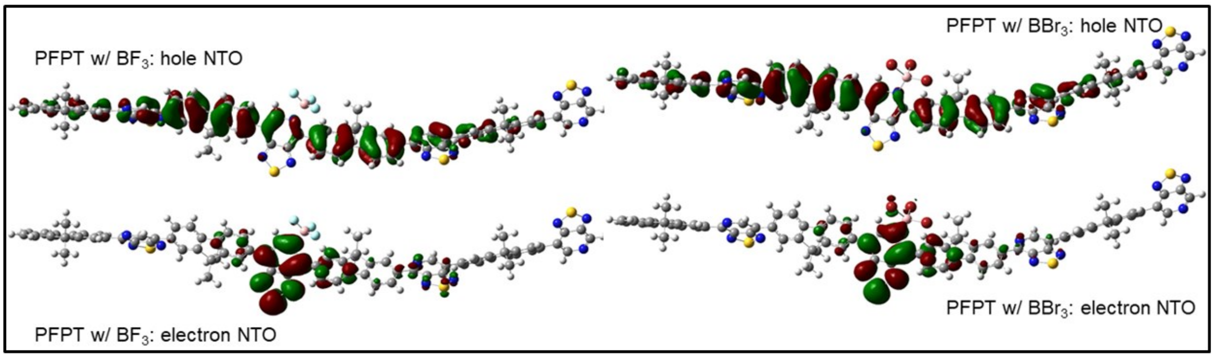


*Figure S3: Lowest electronic excitation natural transition orbitals (NTOs) of the adduct with and $BF_3$ (left) and $BBr_3$ (right).*

*Table S2: Calculated IP, EA and transport gap $E_{gap}$ (in eV) for the neat PFPT tetramer and octamer and for their Lewis acid-base (LAB) adducts with BCF. Excitation wavelength (in nm), energy (in eV) and oscillator strength (f) of the lowest electronic transition $S_0$-$S_1$ are also reported.*

| | **IP** | **EA** | **$E_{gap}$** | **$E(S_0\text{-}S_1)$** | **$f(S_0\text{-}S_1)$** |
|---|---|---|---|---|---|
| **PFPT tetramer** | 5.43 | 2.62 | 2.81 | 546 / 2.27 | 2.23 |
| **PFPT octamer** | 5.37 | 2.66 | 2.71 | 557 / 2.23 | 4.85 |
| **tetramer w/ BCF** | 5.57 | 3.01 | 2.56 | 607 / 2.04 | 0.92 |
| **octamer w/ BCF** | 5.43 | 3.01 | 2.42 | 610 / 2.03 | 1.11 |

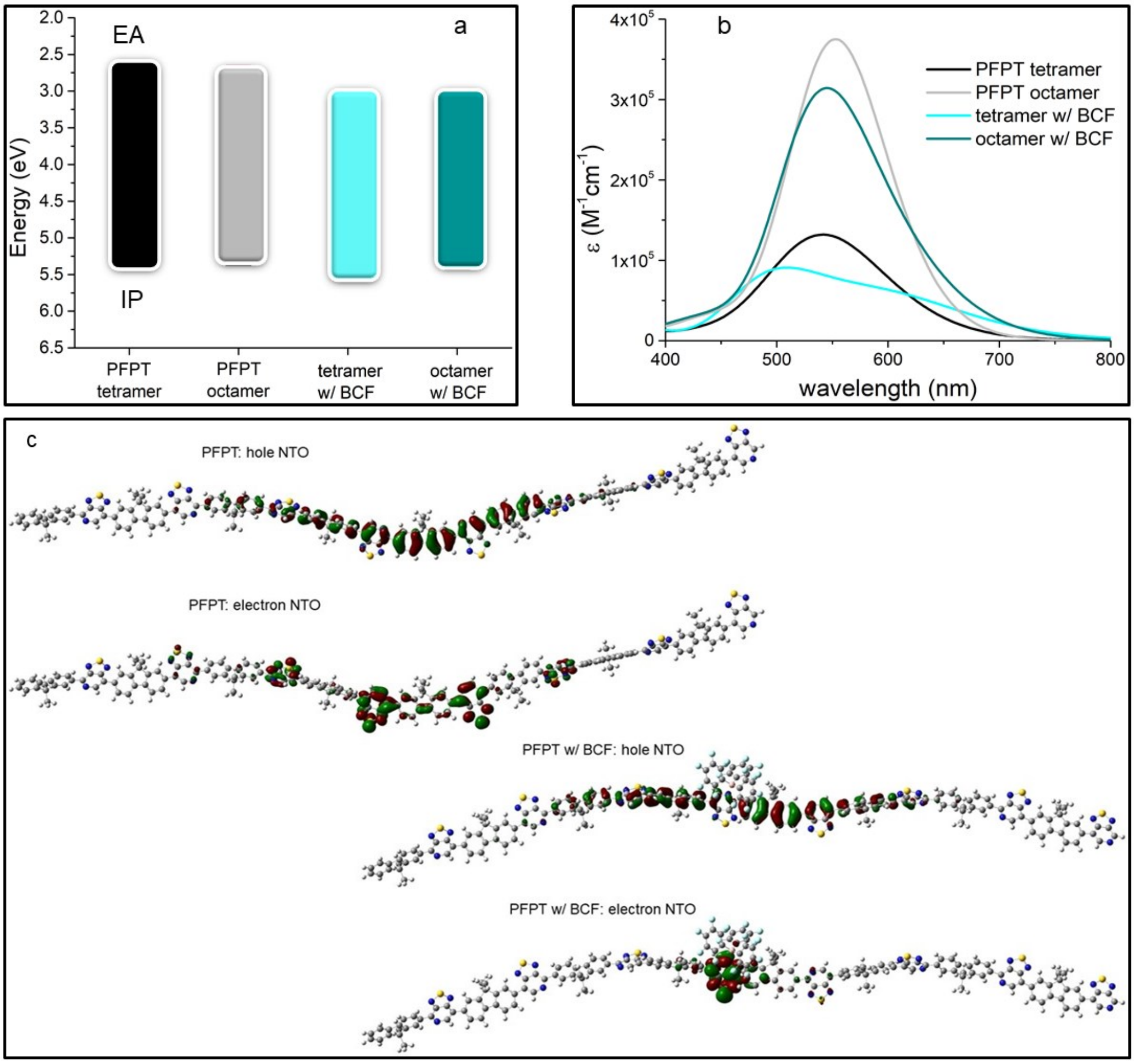


*Figure S4: Energetic diagram showing IP and EA (in eV), b) calculated TD-DFT optical absorption spectra (in nm) for the different species and c) lowest electronic excitation NTOs of the neat PFPT octamer and the adduct with BCF. In panel b) absorption spectra were convoluted with a Full Width Half Maximum of 0.2 eV and the molar absorption coefficient ε is reported on the y-axis.*

## II. PCPDTPT

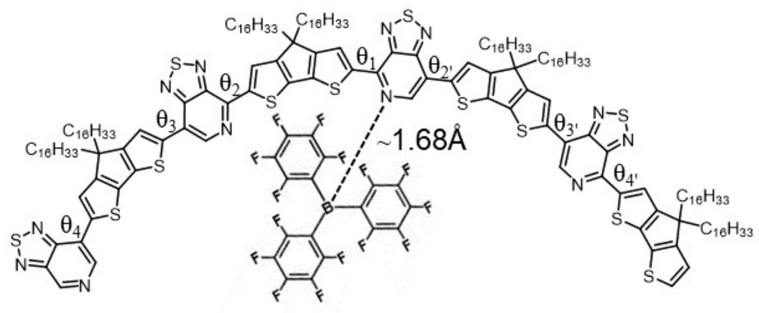


*Figure S5: Chemical structure of the PCPDTPT tetramer bound to BCF.*

*Table S3: Dihedral angles of the neat PCPDTPT tetramer and the different adducts.*

| | $\theta_1$ | $\theta_2$ | $\theta_{2'}$ | $\theta_3$ | $\theta_{3'}$ | $\theta_4$ | $\theta_{4'}$ |
|---|---|---|---|---|---|---|---|
| **Neat** | 0 | 0 | 0 | 0 | 0 | 0 | 0 |
| **w/ $BF_3$** | 39 | 0 | 5 | 12 | 13 | 11 | 0 |
| **w/ BCF** | 112 | 0 | 22 | 14 | 14 | 11 | 0 |
| **w/ $BBr_3$** | 46 | 0 | 21 | 12 | 14 | 11 | 0 |

We performed a ground-state CM5 charge population analysis for the neat PCPDTPT oligomer and when bound to LAs. As in PFPT, the PT group in the neat form carries a slightly negative partial charge (-0.10|e|) and it acts as a LB within the coordinated complex, bearing a positive partial charge of about +0.22|e|. Therefore, the adduct features a partial CT, with the LA molecule carrying a negative partial charge of -0.46|e|. In this case, the boron atomic orbital contribution to the LUMO of the adduct is significantly smaller for $BF_3$ (~0.1%) compared to BCF (~3.5%) and $BBr_3$ (~2.3%).

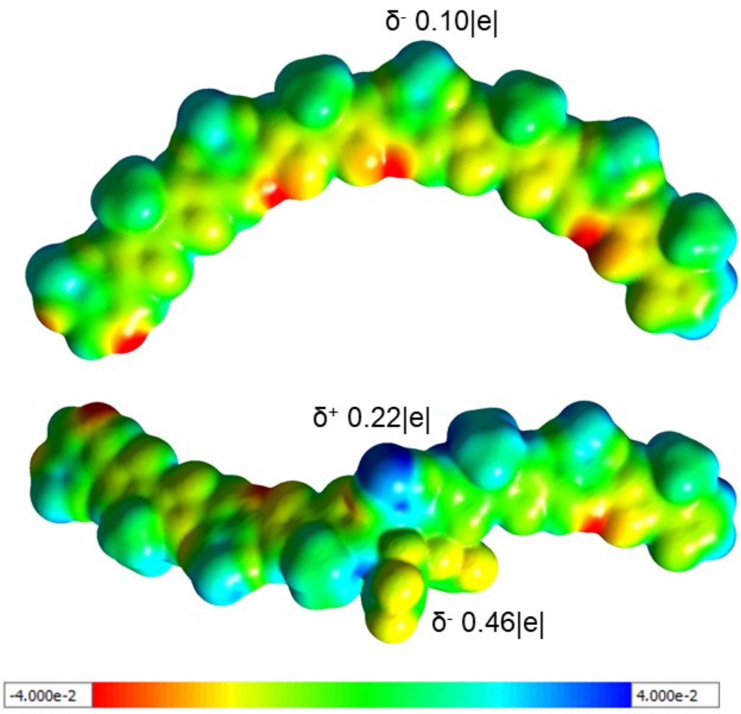


*Figure S6: CM5 charge distribution of the neat tetramer (top) and the adduct with BCF (bottom).*

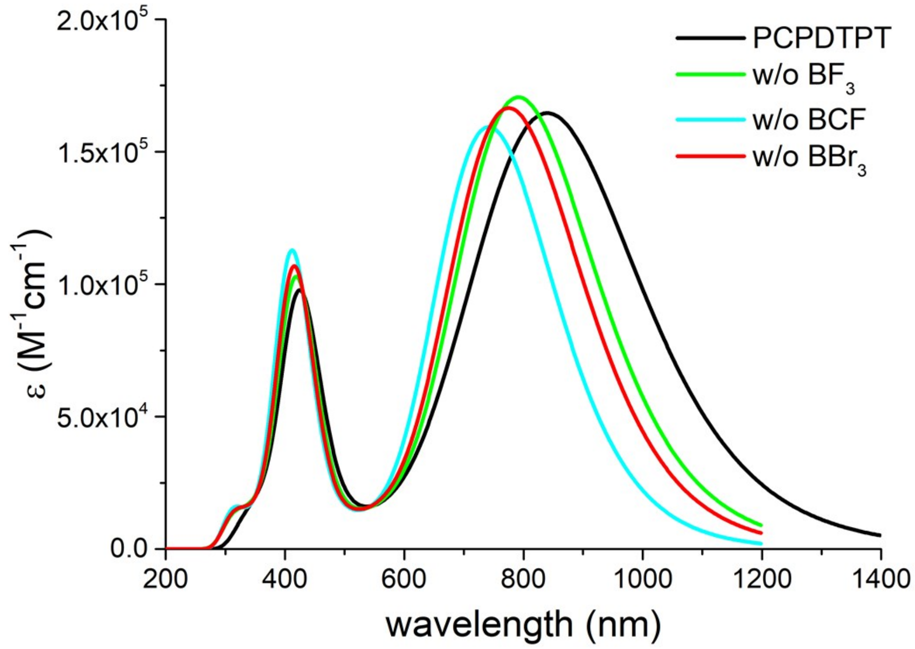


*Figure S7: Calculated TD-DFT optical absorption spectra without the LA. Absorption spectra were convoluted with a Full Width Half Maximum of 0.2 eV and the molar absorption coefficient ε is reported on the y-axis.*

*Table S4: Calculated $E_{gap}$ (in eV), excitation wavelength (in nm), energy (in eV) and oscillator strength (f) of the lowest electronic transition $S_0$-$S_1$ for the pristine PCPDTPT tetramer without the different LAs.*

| | $E_{gap}$ | $E(S_0-S_1)$ | $f(S_0-S_1)$ |
|---|---|---|---|
| **PCPDTPT** | 1.84 | 864 / 1.43 | 2.68 |
| **w/o $BF_3$** | 1.97 | 805 / 1.54 | 2.82 |
| **w/o BCF** | 2.12 | 750 / 1.65 | 2.69 |
| **w/o $BBr_3$** | 2.01 | 789 / 1.57 | 2.70 |

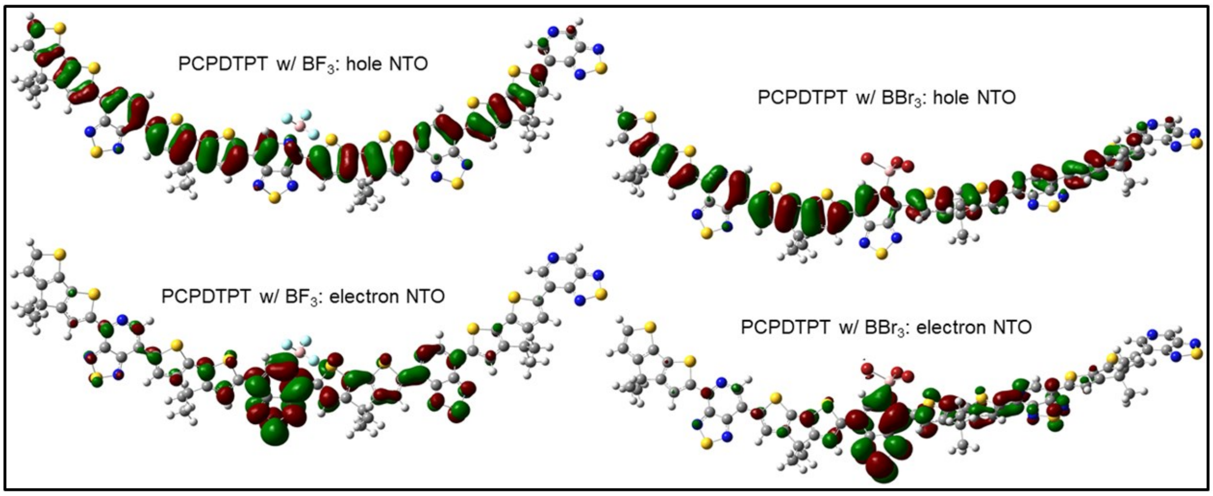


*Figure S8: Lowest electronic excitation natural transition orbitals (NTOs) of the adduct with and $BF_3$ (left) and $BBr_3$ (right).*

## III. PCPTDTBT

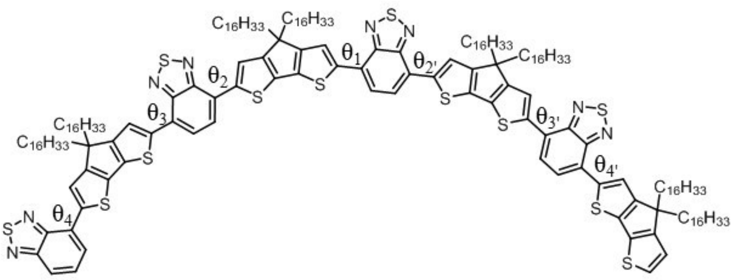


*Figure S9: Chemical structure of the PCPDTBT tetramer.*

*Table S5: Dihedral angles of the neat PCPDTBT tetramer, its radical cation, the protonated tetramer and its radical.*

| | $\theta_1$ | $\theta_2$ | $\theta_{2'}$ | $\theta_3$ | $\theta_{3'}$ | $\theta_4$ | $\theta_{4'}$ |
|---|---|---|---|---|---|---|---|
| **PCPDTBT** | 18 | 17 | 16 | 19 | 20 | 21 | 18 |
| **(PCPDTBT)$^{+\cdot}$** | 18 | 17 | 16 | 19 | 20 | 21 | 18 |
| **(PCPDTBT-H)$^{+}$** | 6 | 21 | 10 | 19 | 53 | 20 | 17 |
| **(PCPDTBT-H)$\cdot$** | 6 | 18 | 0 | 19 | 160 | 21 | 19 |

*Table S6: Computed electronic enthalpy* $\Delta H^{0}_{elec}$ *values for the reactions presented in Scheme 1 and Scheme 2 in the main text, either by modelling species based on a PCPDTBT tetramer or those based on a PCPDTBT octamer.*

| $\Delta H^{0}_{elec}$ | ***w/ PCPDTBT tetramer*** | | ***w/ PCPDTBT octamer*** | |
|---|---|---|---|---|
| | *Protonation* | *Electron transfer* | *Protonation* | *Electron transfer* |
| Scheme 1 | +22.9 | +18.2 | +23.5 | +17.5 |
| Scheme 2 | +36.9 | | +37.0 | |

To check whether the PCPDTBT polaron and the "protonated radical" could in principle be distinguished in the same way, we computed the g-tensor values of the two PCPDTBT radical species. It turns out that the isotropic average g value, $g_{iso}$, of the radical $(PCPDTBT)^{+\cdot}$ cation and the protonated radical (PCPDTBT-H)· species are very different. Namely, for the former, we obtained $g_{iso} = 2.0019$, while for the latter $g_{iso} = 2.0031$. We note that such a difference in g-tensor relates to different spin density distributions being delocalized over the entire molecular backbone in the radical $(PCPDTBT)^{+\cdot}$ cation but more spatially confined in the protonated radical (PCPDTBT-H)·.

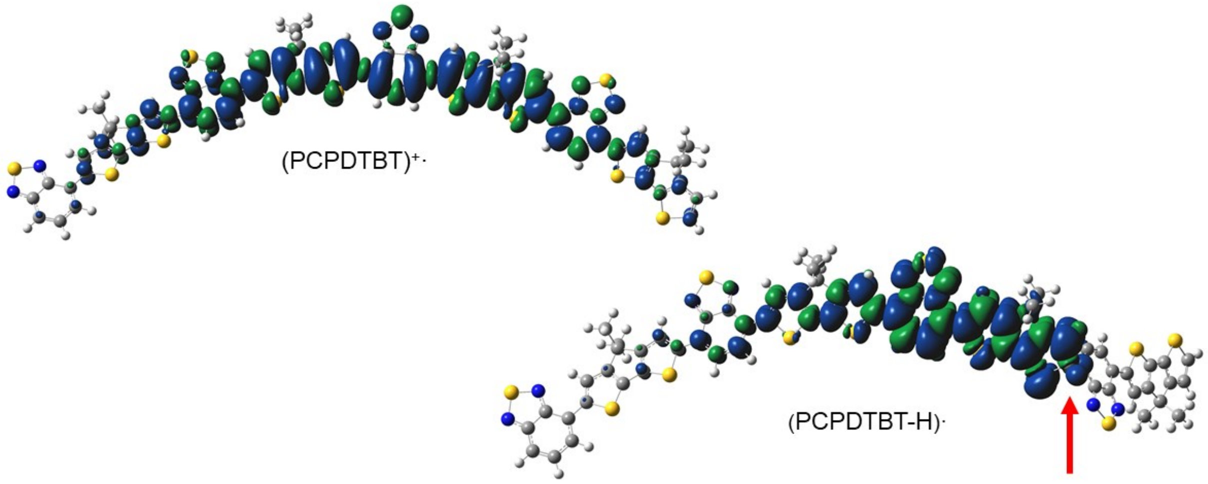


*Figure S10: Spin density distribution of the two radical species. The red arrow point to the position in which the hydrogen atom was added, i.e. an α-carbon atom in the CPDT group.*

## IV. BCF complexes with $H_2O$

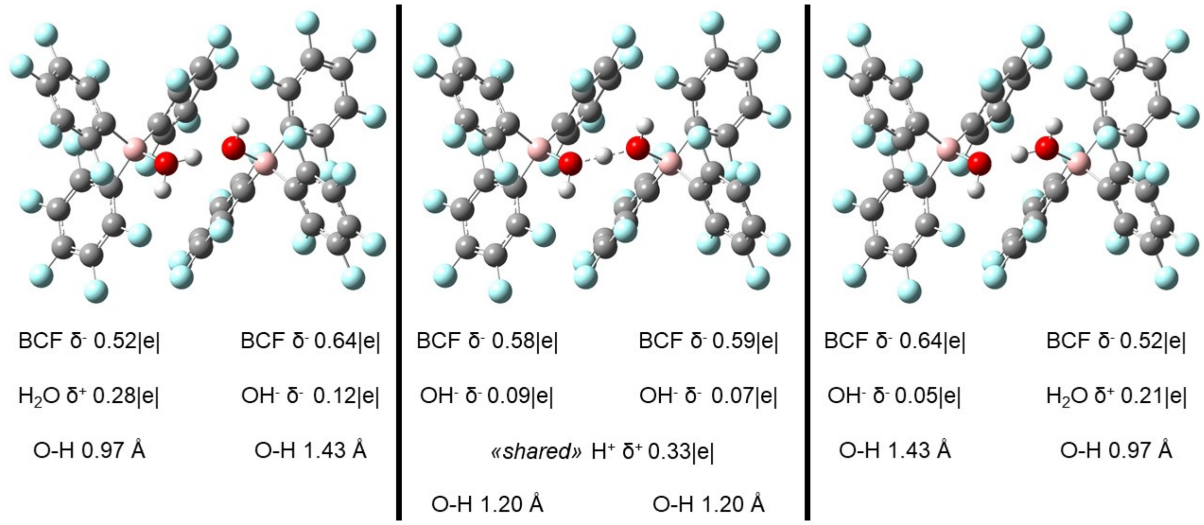


*Figure S11: CM5 charge distributions of the anionic [BCF(OH)(OH$_2$)BCF]$^-$ complex. Here, we changed the interatomic O-H distance, moving the hydrogen atom from the left oxygen to the right one. Note that none of the three cases corresponds to the global minimum, where the H atom is distant 1.10 Å from the O atom (on the left) and 1.30 Å from the other O atom (on the right). The relative energy of the three scenarios with respect to the global minimum is: +0.14 eV (case on the left), +0.02 eV (case in the middle), +0.26 eV (case on the right).*

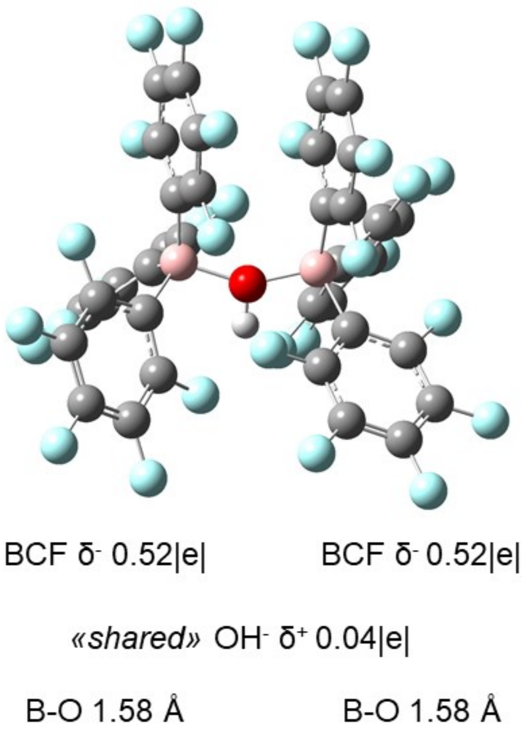


*Figure S12: CM5 charge distributions of the anionic [BCF(OH)BCF]⁻ complex.*

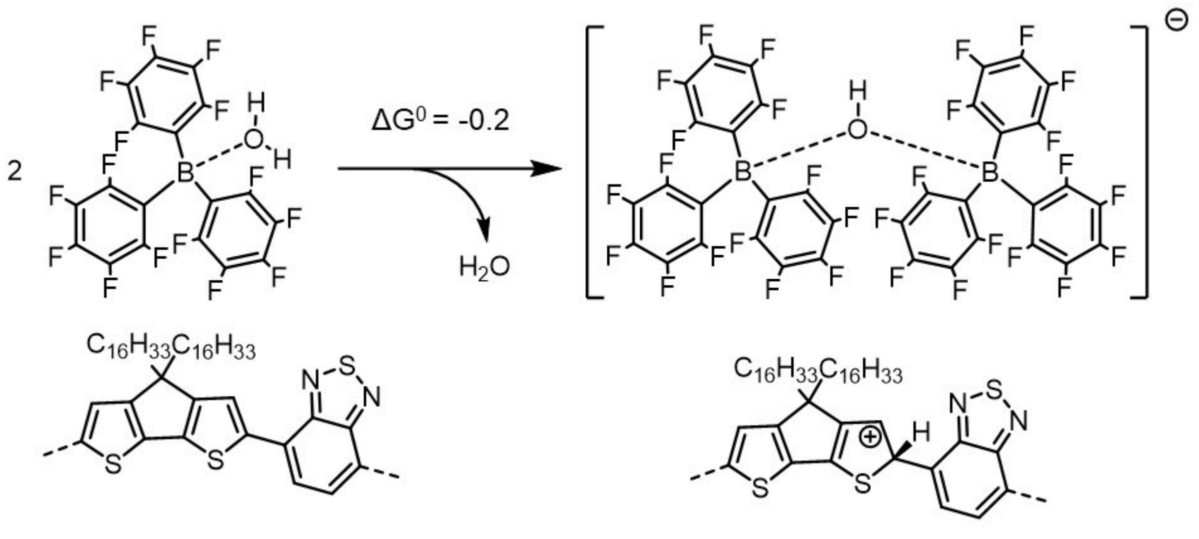


*Figure S13: Formation of the [BCF(OH)BCF]⁻ anion and protonation of the neat PCPDTBT tetramer, when two BCF:OH$_2$ complexes react together and an $H_2O$ molecule is eliminated. DFT calculations yield $\Delta G^0$ = -0.2 kcal mol$^{-1}$ (or -9 meV) for this reaction.*
